# Association between constitution, medical history, axiography and postural control in women aged between 21 to 30 years

**DOI:** 10.1038/s41598-019-56681-8

**Published:** 2019-12-27

**Authors:** C. Doerry, V. Fisch, S. Schamberger, S. Kopp, C. Erbe, E. M. Wanke, D. A. Groneberg, D. Ohlendorf

**Affiliations:** 1 Institute of Occupational Medicine, Social Medicine and Environmental Medicine, Goethe-University Frankfurt/Main, Theodor-Stern-Kai 7, Building 9A, 60596 Frankfurt/Main, Germany; 2 School of dentistry, department of orthodontics, Goethe-University Frankfurt/Main, Theodor-Stern-Kai 7, Building 29, 60596 Frankfurt/Main, Germany; 3 University medical center of the Johannes Gutenberg University Mainz, Augustusplatz 2, 55131 Mainz, Germany

**Keywords:** Health care, Medical research, Epidemiology

## Abstract

The aim of this study was to determine association between constitutional, medical history and axiographic parameters with postural control parameters. Overall, 106 healthy female subjects aged between 21 and 30 years were measured. Data collection was carried out by completing a questionnaire on constitutional parameters, illnesses, accidents and medical/orthodontic therapies, as well as by axio- and posturographic measurements. Data were analyzed using correlations, pair comparisons and group comparisons. The significance level was set at p ≤ 0.05. The statistical evaluation showed significant correlations between sporting exercise and body sway in the sagittal direction (p ≤ 0.03), the BMI and the load on the forefoot/rear foot (p ≤ 0.01), the mouth opening and the load on the forefoot/rearfoot (p ≤ 0.01) and the presence of a deviation with the load on the left/right foot (p ≤ 0.01). The physical condition as well as the temporo-mandibular system are associated with the postural control in young women. Therefore, a holistic diagnosis and therapy will be supported by the present outcomes.

## Introduction

Postural coordination is a multi-segmental product: it depends on physiological processes such as breathing, heartbeat, and imperfect proprioception. Factors causing oral breathing, such as large tonsils or allergies, can also effect the head and body posture. Likewise, psychological aspects, such as mood (for example anxiety), may play a role^1–5^. Total nasal obstruction can also lead to a change in head position^3,6^. Furthermore, it is assumed that mouth breathing can be performed more easily with the head inclined dorsally, since the airways are widened in this position. Head posture, in turn, is functionally anatomically directly related to the whole body posture^7–9^. Besides anamnestic factors, the influence of sporting exercise on postural control is also discussed in many cases: a significant reduction of body sway was observed after several months of exercise^10,11^. In addition, a stronger somatosensory and visual reorganization, specific neural adaptation mechanisms can be further reasons. These specific neural adaptation mechanisms are due to strength training in the claimed muscles and are lead back to intra- and intermuscular coordination processes. Maintaining a constant weight distribution is thus easier for athletes than for non-athletes, because a smaller body sway can be registered^12–14^.

In the literature it will be discussed controversially whether the temporo-mandibular system is associated with postural control^15–28^. In his review, Perinetti^29^ analyzed various studies dealing with the influence of the temporo-mandibular system on postural control. A deviation of the normal function of the temporo-mandibular system is described as temporo-mandibular dysfunction (TMD). The Society for Dental Health, Function and Aesthetics^30^ reports that nowadays about 20% of the population is suffering from a TMD requiring treatment, whereas even a third are affected by TMD-typical parafunctions. TMD patients often suffer from tooth, muscle, temporo-mandibular, ear, head or back pain as well as tinnitus and dizziness. Temporo-mandibular dysfunction can be attributed to dentogenic, myogenic, psychogenic and arthrogenic causes^30–33^. According to several authors^1,34–40^, a TMD is supported by a genetic predisposition, negative psychosocial influences (e.g., stress, anxiety, depression), local factors (e.g., dental occlusion), or dental therapy (e.g., prosthodontic/restorative/orthodontic treatments).

The relationship between the components of the equilibrium - the labyrinth, the eye, and bodily sensibility - on the one hand, and the temporomandibular system on the other hand, has not yet been adequately investigated^16,41–44^. Related to this context, different theories exist for the explanation of possible interdependencies, such as a stimulus propagation through fascia systems ^45,46^ or muscle loops^47^. Also the role of the trigeminal nerve and a possible correspondence of its nuclei with the nuclei vestibulares were discussed^41,48,49^.

The present study was conducted to investigate the influences of the constitution, medical history and axiography on postural control in young healthy women aged between 21–30 years since gender-specific differences in pain threshold, hormonal balance and connective tissue have already been identified^50–52^. Therefore, the focus on one gender—female sex—seems advisable. Since a deterioration of postural control with increasing age was already proven^53,54^, a young healthy test group between the ages of 21 and 30 was selected. In order to make statements about the extent to which impairments of postural control (e.g. after a stroke) in connection with constitution, medical history or axiography can affect postural control, it is first necessary to collect data from subjects who subjectively feel healthy at the time of measurement. A gender comparison is an interesting question after both genders have been analysed.

Therefore, the following hypotheses will be tested:

1. Constitutional and anamnestic parameters influence the body weight distribution in frontal or sagittal direction.
2. Anamnestic parameters such as diseases, pain and allergies lead to an increase in body sway.
3. Regular sporting activities lead to a decrease in body sway.
4. The extent of the mandibular movements differs with the percentage of body weight distribution in the frontal or sagittal dimension.
5. The extent of mandibular movements and deviations of the physiological jaw mobility are related to body sway.

## Material and Methods

### Subjects

**Figure 1:**
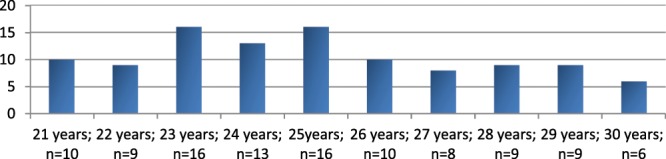
Age distribution of the subjects.

In this study enrolled were 106 healthy adult women aged between 21 and 30 years (25.05 ± 2.68 years) without ongoing anamnesis (Fig. 1). “Healthy“ means that the subjects have no acute symptoms and subjectively described themselves as healthy at the time of measurement.

At the beginning of the measurements a detailed medical history was obtained. Therefore, the function questionnaire of the Department of Orthodontics of the Goethe University Frankfurt/Main (Germany) was used^55^. In addition, information on illnesses, accidents and operations, the subjects were asked to provide further information about orthodontic therapy, their sporting activities as well as constitutional parameters such as body size and weight. The average BMI of the subjects was 21.1 ± 2.61 kg/m², with 6.6% (n = 7) being underweight (<18.5 kg/m^2^) according to the WHO classification, 87.7% (n = 93) were normal (18.5 to 24.99 kg/m^2^) and 5.7% (n = 6) of the subjects were (pre) obese (≥25 kg/m^2^) [30]. Regarding the social status, 72.6% of the subjects were female students and 27.4% belonged to various professional groups (for example, PhD- students, dentists, dental assistants, nurses, office employees, teacher). Many of the participants were dental workers such as dentistry students, dentists or dental assistants.

Excluded from the study were those subjects who reported acute severe pain or current illness or a physically malposture, like a scoliosis. Surgeries on spine, shoulders or pelvis should date back longer than two years. Furthermore, subjects were excluded if they attend physiotherapeutic or orthopedic therapy or take muscle relaxants at the time of measurements.

The rights of these subjects were protected and they were thoroughly familiarized with the study design before giving written informed consent to participate in this study, which was approved by the local ethics committee of the medical faculty of the Goethe-University (Nr. 103/ 16) in accordance with the 1964 Helsinki Declaration and its later amendments.

### Measurement systems

#### Axiography

In order to register electronically various mandibular movements, an axiography was carried out by the Jaw Motion Analyzer (Zebris Medical GmbH, Isny, Germany). The three-dimensional recording of the movements is based on a sonic measurement of ultrasonic pulses between the ultrasound transmitters (attached to the lower jaw) and the recording microphones (fixed on the upper head). The measuring frequency is 50 Hz and the measuring accuracy is 0.1 mm^56–58^. A para-occlusal steel attachment was fixed to the lower jaw with Luxabite (DMG Chemisch-Pharmazeutische Fabrik GmbH, Hamburg, Germany) in order to perform the function analysis.

#### Posturography

The pressure measurement platform GP MultiSens (GeBioM GmbH, Münster, Germany) was used to measure the postural control with a measuring frequency of 100 Hz per sensor (total sampling rate of approx. 500 kHz). The measurement area is 38.5 × 38.5 cm, into which 2304 pressure sensors are integrated. The sensors are arranged in a matrix form and distributed at a density of 1.5 sensors/cm^2^ ^59,60^.

#### Medical history questionnaire

The medical history questionnaire contains questions about: (a) allergies, (b) osteoporosis, (c) rheumatism, (d) diabetes, (e) tinnitus, (f) neurological diseases, (g) headache (h) migraine, (i) pain in joints, (j) pain (k) sound in the temporomandibular joint, (l) pain in the back, (m) accidents on the face (n) accidents on shoulders and/or back and/or pelvis, (o) previous operations, (p) orthopedic therapy, (q) regular drug intake, (r) orthodontic therapy, (s) sporting activity, (t) profession, (u) handedness.

### Examination procedure

#### Axiography

In order to perform the three-dimensional function analysis, a para-occlusal attachment was fixed to the lower jaw. Afterwards, the basic unit and the head arch were fixed to the subject. Jüngling *et al*.^61^ proved a highly valid and reliable method of three-dimensional function analysis. The comparison with a digital caliper shows high efficiency^62^. After calibration and axis determination, all movements of the function module were recorded with double repetition. Subsequently, mean values were calculated from the results of the three measurement repetitions.

The following parameters ware evaluated: (a) maximal laterotrusion to the right/left (mm), (b) maximal protrusion (mm), (c) maximal mouth opening (mm), (d) deviation during mouth opening, (e) deflection during mouth opening.

#### Posturography

Each subject was instructed to stand within the circle depicted on the plate, in habitual body position, jaw in rest position and fixing a point at eye level, without moving as far as possible. The postural control was recorded for 30 seconds with a three-time measurement repetition. The following parameters were used for the statistical evaluation: (a) percentage distribution of the left/right forefoot (%), (b) percentage distribution of left/right rearfoot (%), (c) percentage distribution of left/right foot, (d) percentage distribution of forefoot/rearfoot (%), (e) maximal body sway in frontal direction (mm), (f) maximal body sway in sagittal direction.

### Statistical evaluation

The data was analyzed using the statistics program BiAS 11.03 (Epsilon Verlag, Darmstadt, Germany) [33]. The data was first tested for normal distribution by the Kolmogoroff-Smirnov-Lilliefors test. For normal-distributed data, the Pearson correlation and the sample t-test were used. For non-normalized data, the Spearman correlation, the Kruskal-Wallis-test with post-hoc tests, the Wilcoxon-matched-pairs test, the Wilcoxon-Mann-Whitney-U-test and the Jonckheere Terpstra test for trend were conducted. For the effect strength, the correlation coefficient rho was used according to different classes (Cohen^63^, Rosenthal^64^, Evans^65^). No adjustment for multiple comparison was made. The significance level was set at 5%.

## Results

### Medical history

The mean age of the 106 subjects was 25.05 ± 2.68 years. All women were between 1.5 and 1.82 m tall (1.69 ± 0.06 m) and had a body weight of 46 to 106 kg (60 ± 7.85 kg), resulting in body mass indices between 16.9 and 37.56 kg/m² (21.1 ± 2.61 kg/m²).

The results of the medical history are listed in Table 1.

**Table 1:** Indicated diseases and events in the patient’s medical history ordered by frequency.

Medical history parameter	Number (percentage)
Pain in the back	35 (33.0%)
Allergies	33 (31.1%)
Noises in the temporomandibular joint	32 (30.2%)
Orthopedic therapy	24 (22.6%)
Headache/Migraine	17 (16.0%)
Regular medication intake	17 (16.0%)
Earlier surgeries	16 (15.1%)
Pain in the temporomandibular joint	15 (14.1%)
Other disorders	12 (11.3%)
Pain in the joints	7 (6.6%)
Accidents in mouth, jaw or facial area	4 (3.8%)
Accidents on shoulders, back or pelvis	4 (3.8%)
Tinnitus	1 (0.9%)
Neurological diseases	1 (0.9%)
Diabetes	1 (0.9%)
Rheumatism	0 (0%)
Osteoporosis	0 (0%)

In this context, 33% of participants (n = 35) showed pain in the back, 33.1% (n = 33) showed allergies and 30.2% (n = 32) noises (crackling or grinding sounds) in the temporomandibular joint. 22.6% (n = 24) stated that they had undergone an orthopedic therapy and 16% (n = 17) showed medication or declared that they often suffered from headache/migraine. In addition, 15.1% (n = 16) of the participants already had surgery, 14.1% (n = 15) reported pain in the temporomandibular joint, and 11.3% (n = 12) had other diseases. For all other parameters, less than 10% of the respondents reported positive results. 75.5% (n = 80) of the women reported a completed orthodontic therapy and 86.8% (n = 92) reported regular sporting activity. In addition, 4.7% (n = 5) of the participants were left-handed persons.

### Medical history-postural control

Table 2 includes all correlations between the postural control and the medical history data such as age, height, weight and BMI.

**Table 2:** Testing for correlations with constitutional parameters; significant p values are bold and marked with *.

	Age			Heights			Weight			BMI		
	rho	Effect-size	p-value	rho	Effect-size	p-value	rho	Effect-size	p-value	rho	Effect-size	p-value
Left foot(%)	0.00	poor	0.99	−0.03	poor	0.77	−0.10	poor	0.31	−0.06	poor	0.52
Right foot (%)	0.00	poor	0.99	0.03	poor	0.77	0.10	poor	0.31	0.06	poor	0.52
Forefoot (%)	0.12	poor	0.21	−0.01	poor	0.89	0.16	poor	0.09	0.24	weak	0.01^*^
Rearfoot(%)	−0.12	poor	0.21	0.01	poor	0.89	−0.16	poor	0.09	−0.24	weak	0.01^*^
Frontal sway (mm)	0.09	poor	0.36	0.12	poor	0.23	0.04	poor	0.68	−0.01	poor	0.92
Sagittal sway (mm)	0.14	poor	0.15	0.19	poor	0.05	0.05	poor	0.61	−0.10	poor	0.32

The effect size classification is as follows: (<0.2: poor −0.2–0.4: weak −0.4–0.6: moderate −0.6–0.8: strong − > 0.8: optimal).

There were no correlations between postural control and age, height as well as weight parameters, respectively (p ≥ 0.05). With respect to the BMI, p-values of p ≤ 0.01 are noted for the forefoot/rearfoot distribution. As the BMI increases, the percentage distribution in the “forefoot” increases and the percent distribution in the “rearfoot” decreases with an effect strength of “weak” (average effect after Cohen).

No significant results (p ≥ 0.05) were found when testing possible correlations of the parameters “allergies”, “back pain”, “orthopedic therapy” and “headache/migraine” with the postural control (Table 3).

**Table 3:** Influence of different anamnestic parameters on the postural control; significant p values are bold and marked with *.

	Allergies			Back pain			Orthopedic therapies			headache/Migraine		
	rho	Effect-size	p-value	rho	Effect-size	p-value	rho	Effect-size	p-value	rho	Effect-size	p-value
Left foot (%)	0.04	poor	0.68	0.13	poor	0.18	0.13	poor	0.18	0.07	poor	0.49
Right foot (%)	0.04	poor	0.68	0.13	poor	0.18	0.13	poor	0.18	0.07	poor	0.49
Forefoot (%)	0.02	poor	0.87	0.09	poor	0.38	0.04	poor	0.69	0.06	poor	0.54
Rearfoot(%)	0.02	poor	0.87	0.09	poor	0.38	0.04	poor	0.69	0.06	poor	0.54
Frontal sway (mm)	0.13	poor	0.18	0.09	poor	0.34	0.19	poor	0.05	0.01	poor	0.93
Sagittal sway (mm)	0.16	poor	0.10	0.04	poor	0.70	0.06	poor	0.54	0.04	poor	0.66

The effect size classification is as follows: (<0.2: poor − 0.2–0.4: weak − 0.4–0.6: moderate − 0.6–0.8: strong − >0.8: optimal).

The parameters “pain in the temporomandibular joint” and “sounds in the temporomandibular joint” were also calculated for correlations with the postural control. First, the subjects with pain and/or sounds in the temporomandibular joint were compared with the other participants, who had no such complaints. Afterwards, the different groups were compared among themselves, differentiating between “no pain/sounds”, “right-sided pain/sound”, “left-sided pain/sound” and “bilateral pain/sounds” (Table 4). No significance was found for any of these correlations (p ≥ 0.05).

**Table 4:** Influence of pain and noises in the temporomandibular joint on postural control; distinguished in (1) pain/no pain or noises/no noises and (2) median.

	Pain in the temporo-mandibular joint yes/no			Pain in the temporo-mandibular joint right/left side								
				No pain		Right side pain		Left side pain		Both sides pain		p-value (Kruskal Wallis)
	rho	Effect-size	p-value	Median	Min/Max.	Median	Min/Max.	Median	Min/Max.	Median	Min/Max.	
Left foot (%)	0.07	poor	0.48	50.67	32.33/65.33	47.67	34.00/63.33	43.67	43.33/44.00	49.50	41.67/60.67	0.46
Right foot (%)	0.07	poor	0.48	49.33	34.67/67.67	52.33	36.67/66.00	56.34	56.00/56.67	50.50	39.33/58.33	0.46
Forefoot (%)	0.04	poor	0.71	33.33	18.67/53.67	34.00	29.67/58.33	32.00	28.33/35.67	27.50	22.33/53.67	0.73
Rearfoot(%)	0.04	poor	0.71	66.67	46.33/81.33	66.00	41.67/70.33	68.00	64.33/71.67	72.50	46.33/77.67	0.73
Frontal sway (mm)	0.05	poor	0.63	9.33	3.67/18.33	8.00	5.00/10.33	6.67	2.33/11.00	9.84	4.33/25.00	0.81
Sagittal sway (mm)	0.03	poor	0.79	13.00	4.33/24.67	13.00	8.33/15.33	13.83	7.33/20.33	13.00	6.33/32.00	0.98
	**Noises in the temporo-mandibular joint** **yes/no**			**Noises in the temporo-mandibular joint right/left side**								
				**No noises**		**Right side pain**		**Left side pain**		**Both sides noises**		**p-value (Kruskal-Wallis)**
	**rho**	**Effect-size**	**p-value**	**Median**	**Min/Max**.	**Median**	**Min/Max**.	**Median**	**Min/Max**.	**Median**	**Min**. **/Max**.	
Left foot (%)	0.13	poor	0.19	51.67	32.33/65.33	48.67	38.67/59.67	51.34	45.33/61.67	49.33	33.00/63.33	0.34
Right foot (%)	0.13	poor	0.19	48.33	34.67/67.67	51.33	40.33/61.33	48.67	38.33/54.67	50.67	36.67/67.00	0.34
Forefoot (%)	0.08	poor	0.42	33.50	18.67/53.67	31.67	26.33/52.33	33.17	24.33/44.33	27.33	23.67/58.33	0.43
Rearfoot(%)	0.08	poor	0.42	66.50	46.33/81.33	68.33	47.67/73.67	66.84	55.67/75.67	72.67	41.67/76.33	0.43
Frontal sway (mm)	0.11	poor	0.25	9.17	3.67/18.33	9.67	2.33/25.00	14.00	7.00/16.00	8.33	4.67/13.33	0.09
Sagittal sway (mm)	0.17	poor	0.08	12.17	4.33/24.67	13.67	6.33/32.00	14.33	12.67/20.00	13.67	8.33/15.67	0.29

Minimum and maximum postoperative parameters for the affected temporomandibular joints; significant p values are bold and marked with *. The effect size classification is as follows: ( < 0.2: poor − 0.2–0.4: weak − 0.4–0.6: moderate − 0.6–0.8: strong − > 0.8: optimal).

Other parameters of the medical history questionnaire were the handedness, the frequency of the sporting activity and the presence of completed orthodontic therapy (Table 5). A significant p-value was calculated with p ≤ 0.03 for correlation between sagittal sway and the “frequency of sporting activity”. However, it should be noted that in the successive group comparisons of the subgroups of the parameter “frequency of sporting activity” no significances were calculated. No significances could be found for the “handedness” (p ≥ 0.05).

**Table 5:** Influence of handedness, frequency of sporting exercise and an orthodontic therapy on postural control; significant p values are bold and marked with *.

	Handedness			Frequency of sporting exercise			Orthodontic therapy		
	rho	Effect-size	p-value	rho	Effect-size	p-value	rho	Effect-size	p-value
Left foot (%)	0.12	poor	0.22	—	—	0.1	0.01	poor	0.91
Right foot (%)	0.12	poor	0.22	—	—	0.1	0.01	poor	0.91
Forefoot (%)	0.15	poor	0.12	—	—	0.45	0.16	poor	0.10
Rearfoot (%)	0.15	poor	0.12	—	—	0.45	0.16	poor	0.10
Frontal sway (mm)	0.03	poor	0.75	—	—	0.27	0.01	poor	0.94
Sagittal sway (mm)	0.04	poor	0.72	—	—	0.03*	0.10	poor	0.28

The effect size classification is as follows: (<0.2: poor − 0.2–0.4: weak − 0.4–0.6: moderate − 0.6–0.8: strong − >0.8: optimal). The Jonckheere-Terpstra-Tests auf Trend does not give any information about the rho-value and the effect strength.

### Axiography

**Figure 2:**
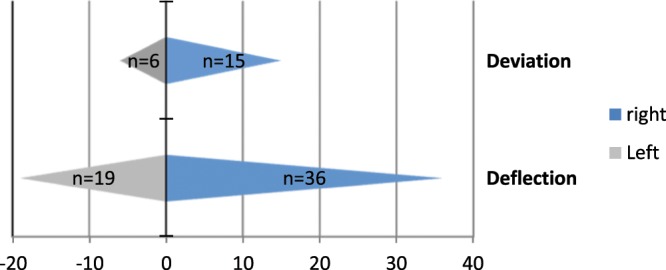
Frequency distribution of the deviation and deflection to the right and left side.

When measuring the mandibular mobility, lateral protrusion to the right and left side, protrusion and mouth opening were recorded (Tables 6 and 7). Thereby, the distance between the mandible and the initial position (habitual occlusion) was determined. Laterotrusion to the right and left as well as protrusion were similar in all subjects. In addition, the mouth opening was measured on average 43.87 ± 5.70 mm. In addition to the mandibular mobility, the presence of a deviation or deflection during mouth opening was also examined (Fig. 2). A deviation was found in 76 subjects (71.7%). Both, the deviation as well as the deflection, could be observed more frequently to the right side.

**Table 6:** Extent of mandibular movements.

	Mean ± SD	Minimum	Maximum
Laterotrusion to the right (mm)	9.70 ± 1.97	4.3	14.9
Laterotrusion tot he left(mm)	9.59 ± 1.86	4.4	14.4
Protrusion (mm)	9.38 ± 1.78	4.0	15.1
Mouth opening (mm)	43.87 ± 5.70	26.6	57.8

**Table 7:** Deviation and deflection during opening with direction and time of the deviation.

	Available at	Direction		direction time		
		Right	Left	Initial	Intermediary	Terminal
Deviation	21 (19.8%)	15 (14.2%)	6 (5.7%)	8 (7.5%)	9 (8.5%)	4 (3.8%)
Deflection	55 (51.9%)	36 (34.0%)	19 (17.9%)	27 (25.5%)	18 (17.0%)	10 (9.4%)

### Axiography- postural control

The calculated p-values for the correlation between laterotrusion to the right and left side, respectively, and the postural control were not significant (p ≥ 0.05) (Table 8).

**Table 8:** Correlation of laterotrusion to the right and left with the parameters of the postural control; significant p-values are bold and marked with *.

	Laterotrusion right			Laterotrusion left		
	rho	Effect-size	p-value	rho	Effect-size	p-value
Left foot (%)	0.14	poor	0.15	0.07	poor	0.46
Right foot (%)	−0.14	poor	0.15	−0.07	poor	0.46
Forefoot (%)	0.14	poor	0.15	0.08	poor	0.44
Rearfoot(%)	−0.14	poor	0.15	−0.08	poor	0.44
Frontal sway (mm)	0.05	poor	0.61	0.08	poor	0.44
Sagittal sway (mm)	−0.05	poor	0.59	0.06	poor	0.56

The effect size classification is as follows: (<0.2: poor − 0.2–0.4: weak − 0.4–0.6: moderate − 0.6–0.8: strong −>0.8: optimal).

In addition to laterotrusion, the maximal mouth opening (in mm) of the subjects was also correlated with the posturography parameters (Table 9). For the forefoot and rearfoot loading p-values of p ≤ 0.01 were determined. The rho-value of 0.24 (Evans: “Weak”) indicates a positive trend for the forefoot load while a negative trend can be seen for the rearfoot load. A wide mouth opening correlates with an increased load on the forefoot and a reduced load on the rearfoot. No significance could be found for protrusion (p ≥ 0.05).

**Table 9:** Correlations of the mouth opening and protrusion with the parameters of the postural control; significant p-values are bold and marked with *.

	Mouth opening			Protrusion		
	rho	Effect-size	p-value	rho	Effect-size	p-value
Left foot (%)	−0.01	poor	0.92	0.03	poor	0.80
Right foot (%)	0.01	poor	0.92	−0.03	poor	0.80
Forefoot (%)	0.24	weak	0.01*	0.01	poor	0.94
Rearfoot(%)	−0.24	weak	0.01*	−0.01	poor	0.94
Frontal sway (mm)	−0.05	poor	0.63	0.00	poor	0.98
Sagittal sway (mm)	−0.06	poor	0.51	−0.08	poor	0.42

The effect size classification is as follows: (<0.2: poor − 0.2–0.4: weak − 0.4–0.6: moderate − 0.6–0.8: strong − >0.8: optimal).

The following medians were calculated for the load distribution of the left foot: 54.17% for subjects with deviation to the left, 51.67% for subjects without incisal point deviation and 49.33% for subjects with a deviation to the right (Table 10). Accordingly, the load distribution of the right foot averaged 45.84%, 48.33% and 50.67%, respectively. Significant p-values of p ≤ 0.01 were determined for the load on the left/right foot.

**Table 10:** Median, minimum and maximum of the posturometric parameters for the different directions of deviation; Comparison between the group “deviation” and “no incisal point deviation” with respect to the variation; significant p values are bold and marked with *.

	Deviation to the left		No incisal point deviation		Deviation to the right		p-value
	Median	Min/Max.	Median	Min/Max.	Median	Min/Max.	
Left foot (%)	54.17	50.00/64.33	51.67	33.00/57.33	49.33	32.33/65.00	0.01*
Right foot (%)	45.84	35.67/50.00	48.33	42.67/67.00	50.67	35.00/67.67	0.01*
Forefoot (%)	35.67	24.67/38.33	33.17	21.33/50.67	33.33	22.33/53.67	0.47
Rearfoot (%)	64.34	61.67/75.33	66.84	49.33/78.67	66.67	46.33/77.67	0.47
	**Deviation**			**No incisal point deviation**			**p-value (Wilcoxon)**
	**Median**	**Min/Max**.		**Median**		**Min/Max**.	
Frontal sway (mm)	10.33	4.67/25.00		9.67		3.67/18.33	0.63
Sagittal sway (mm)	12.67	7.00/32.00		13.84		5.00/24.67	0.67

Neither significant correlations with the presence of a deviation (p ≥ 0.05) were found for the percentage load distribution of the forefoot and rearfoot, nor for the frontal or sagittal body sway. There were also no significances (p ≥ 0.05) for the effects of the deflection direction on postural control (Table 11).

**Table 11:** Median, minimum and maximum of the posturometric parameters for the different directions of the deviation; Comparison between the group “deviation” and “no incisal point deviation” with respect to the variation; significant p values are bold and marked with *.

	Deflection to the left		No incisal point deviation		Deflection to the right		p-value
	Median	Min/Max.	Median	Min/Max.	Median	Min/Max.	
Left foot (%)	50.67	39.00/61.67	51.67	33.00/57.33	48.34	34.00/65.33	0.12
Right foot (%)	49.33	38.33/61.00	48.33	42.67/67.00	51.67	34.67/66.00	0.12
Forefoot (%)	29.67	18.67/46.33	33.17	21.33/50.67	33.50	23.67/58.33	0.10
Rearfoot (%)	70.33	53.67/81.33	66.84	49.33/78.67	66.50	41.67/76.33	0.10
	**Deflection**			**No incisal point deviation**			**p-value (Wilcoxon)**
	**Median**	**Min/Max**.		**Median**	**Min/Max**.		
Frontal sway (mm)	7.33	2.33/16.33		9.67	3.67/18.33		0.05
Sagittal sway (mm)	12.33	4.33/21.00		13.84	5.00/24.67		0.09

## Discussion

In this study associations between constitution, axiography and postural control could be found. With increasing BMI the weight distribution shifts on the forefoot. Sagittal body sway also decreases with regular physical activity. Most axiographical parameters did not show correlations with postural control parameters. However, it can be noted that the further the mouth can be opened, the more forefoot load can be seen. No correlations between medical history of the subjects with postural control could be detected.

The percentage load redistribution from rear foot to forefoot (low effect strength) with increasing BMI may be due to the fact that there is more body mass in the chest/abdominal region in subjects with a higher BMI and therefore the Center of Pressure (CoP) in these subjects drift anteriorly. In this context, an association between an above-average BMI and an increased load on the forefoot could already be demonstrated^66,67^. No correlations with the postural control could be found for all other constitutional parameters (age, height and weight). Other studies showed that age influenced the balance^53,54^. Since all subjects in this study had a similar age (21–30 years) this influence could not be demonstrated.

In principle, the mean BMI (21.1 kg/m²) of the participants was 2.6 kg/m² below the data of Mensink *et al*.^68^ for the same age group (18 to 29 years, 23.7 kg/m^2^), which is attributable to larger and lighter participants (169 cm to 165.8 cm, 60.28 kg to 65.2 kg)^68^.

In 2009, the Federal Statistical Office^69^ raised an average size of 168 cm, an average weight of 63.5 kg and an average BMI of 22.65 kg/m² for the age group of the 20–30-year-old women. These data are more similar to the present results than those of Mensink *et al*.^68^. One possible reason for these deviations is the social status of the subjects^68^, which can be assumed as outstanding for academics. The weight difference could also lead back to an academic study in order to a more conscious diet as well as a high stress level. However, weight and body size are based on subjective data of the subjects, so that possible misstatements have to be taken into consideration.

Basically, back pain did not affect the postural control, although it has to be considered that no one suffered from severe back pain. Menz *et al*.^70^ confirmed this statement since lower back pain does not affect the foot position. This also applies to the parameters “pain in the jaw joint” and “sound in the jaw joint”. Although 14% and 30% of the subjects confirmed these parameters, they were not able to define them as a severe TMD.

The complaints of the subjects were classified to the definition of Helkimo^71^. It is useful to consider in future analysis TMD classifications of other authors^1,33–39,72–75^, too. The fact that none of the subjects suffered from a severe TMD is possibly the reason why, unlike other studies, no correlations could be found between jaw joint complaints and Centre of Pressure. Also, the young age of the subjects may play a role: It is possible that a myogenic caused temporo-mandibular dysfunction manifests itself only after a prolonged presence in a body weight load distribution. This body weight load distribution results in an anteriorly inclined head posture, which cause a shortening of the posterior cervical extensors as well as the M. Sternocleidomastoideus^2,7^. Thus, the cervical lordosis is strengthened by an inclination of the head as a countermovement. An inclination of the head also results in an anterior shift of the Centre of Pressure: the body weight of the forefoot increased^7,76–78^.

The present results show that the frequency of sporting exercise has a significant effect on the sagittal body sway. A significant reduction of body sway occurs after several months of exercise^10,11^, whereas Zemková *et al*.^11^ explicitly describe a change in the sagittal sway direction.

In addition to a stronger somatosensory and visual reorganization, specific neural adaptation mechanisms are possible reasons. These processes develop through strength training in the claimed muscles and are lead back to the intra- and intermuscular coordination. Maintaining a constant weight distribution is easier for athletes than for non-athletes. Therefore, athletes have a lower body sway^12–14^.

On the other hand, neither the handedness nor the completed orthodontic therapy have significant effect on postural control in young women. If, however, the mean values for the right and left loads are viewed separately for right- and left-handers, right-handedness predominates in the right foot and in left-handers predominates in the left foot, too. This correlation is not significant due to the small number of left-handed persons (n = 5). On the basis of the widespread assumption that right-handed persons are at the same time the right-footed, several authors have found that the handedness does not always coincide with the footedness^79–81^, since each person has one body-side more coordinate-motor pronounced.

The origin of commands for a fine-motor skilled hand or foot movement is localized for right-handed persons in the left hemisphere due to a contralateral hemispheric intersection^82^. In addition to favoring body side in terms of handedness and footedness, a favored chewing side could also be confirmed^83^. In this study the women were born between 1985 and 1995. They showed a ratio of 95.3% right-handers to 4.7% left-handers. According to Bublak^84^, this is a similar distribution as in the 1970s, when about 90% of the population was right-handed and right-footed at the same time. Of the remaining 10%, only half were left-footed, the other right-footed. A social conscious or subconscious re-training of the left-handers could be the reason^84^. Even the assumption that the handedness affects the footedness is not sufficient for an increased displacement of the body weight to the right or to the left, since the footedness can be determined not by load measurement, but by different tests^85^. For a reliable statement about the relationship between handedness and the Center of Pressure shift studies with a larger proportion of left-handers are necessary in future investigation. However, it is difficult due to the low amount of left-handers in Germany (10–15%^86^).

In the present study, 75.5% of the patients underwent orthodontic therapy. It would be useful to calculate correlations between the Center of Pressure and subjects who neither had orthodontic nor other dental treatments, since this result in subjects with original occlusion. Unfortunately, nowadays it is difficult to find such a collective of suitable size and age (21 to 30 years). Also, an even distribution of athletes and non-athletes would ensure a better comparison of the two groups. In this study, 86.8% of the subjects reported regular exercise.

According to an exercise study carried out by the health insurance company “Techniker Krankenkasse” ^87^ in 2016, 48% of the population never, or rarely, perform regularly physical exercises. The frequent exercise of the subjects of this study may be due to social status and young age.

The analysis of the axiography revealed no effects of laterotrusion and protrusion on the percentage body weight load distribution. The mouth opening correlated significantly with the percentage body weight distribution of the forefoot and rear foot. Subjects who had a wide mouth opening were more prominent on the forefoot than those who could open their mouths less widely. In this context, the influence of head retention has to be taken into account since posterior head tilting can lead to an increased load on the rear foot and at the same time to a growth inhibition of the upper and lower jaw^88^.

There is a relation between increased extension of the cervical spine and reduced upper and lower jaw, which may also cause a reduced mouth opening. A large mandible causes a greater incisal distance to the upper jaw than a smaller mandible at the same opening angle. On the basis of this theory, a correlation of the mouth opening with the forefoot load is obvious. Authors^89,90^ who investigated the mouth opening width and the head position came to the conclusion that the incisal distance is greater in an anterior tilted head position than in the neutral position. With dorsal inclination of the head, the subjects could open the mouth the least. This phenomenon can be explained by the interactions of chewing and throat musculature as well as by varying condylar movements^89,90^. Therefore, it could be concluded that subjects with a wide mouth opening are naturally characterized by a pre-determined head posture, which is noticeable in the posturographic analysis by a larger load on the forefoot resulting from the anterior displacement of the Center of Pressure.

It appears useful to correlate the asymmetry of muscular activity due to a preferred chewing side as well as to the head position in future studies, since no significant correlations could be demonstrated for the deflection.

This group of subjects investigated may not represent in total the 21 to 30-year-old women in Germany. Since many of the participants were dental workers such as dentistry students, dentists or dental assistants, they often have an unhealthy posture during their work, which often leads to back pain^91,92^. It cannot be ruled out that these errors are already present to a small extent in students and has an effect on the postural control of the subjects. However, all subjects felt subjectively healthy and were able to carry out the surveys as well as respond adequately to instructions from the treatment providers, thus ensuring the best possible data collection. It has also been considered that this study is cross-sectional in nature so that causalities cannot be determined, In principle, it must also be taken into account that only women aged 21–30 years were measured in this study. It would be interesting to find out in further studies with equal inclusion and exclusion criteria to what extent these results differ from those of women of different ages (31+ years) but also from those of men.

## Conclusion

Although the results of the present study do not show any correlation with a percentage body weight load distribution for most constitutional and anamnestic data, the influence of the body mass index on the body weight distribution in the sagittal direction from the rear foot to the forefoot could be found. Sagittal body sway also decreases with regular physical activity. Participants with an increased load on the forefoot have an enlarged mouth opening, but no change neither in protrusion nor laterotrusion. Nevertheless, a correlation between the load on the left/right foot and the presence of a deviation could be found.
